# Reinforcement-Learning-Based Fixed-Time Prescribed Performance Consensus Control for Stochastic Nonlinear MASs with Sensor Faults

**DOI:** 10.3390/s24247906

**Published:** 2024-12-11

**Authors:** Zhenyou Wang, Xiaoquan Cai, Ao Luo, Hui Ma, Shengbing Xu

**Affiliations:** 1 School of Mathematics and Statistics, Guangdong University of Technology, Guangzhou 510006, China; zywang@gdut.edu.cn (Z.W.); xiaoquancai6@gmail.com (X.C.); huima2016@163.com (H.M.); xushengbing@gdut.edu.cn (S.X.); 2 School of Automation, Guangdong-Hong Kong Joint Laboratory for Intelligent Decision and Cooperative Control, Guangdong University of Technology, Guangzhou 510006, China

**Keywords:** fixed-time prescribed performance, optimal consensus control, reinforcement learning, sensor faults, stochastic nonlinear multi-agent systems

## Abstract

This paper proposes the fixed-time prescribed performance optimal consensus control method for stochastic nonlinear multi-agent systems with sensor faults. The consensus error converges to the prescribed performance bounds in fixed-time by an improved performance function and coordinate transformation. Due to the unknown faults in sensors, the system states cannot be gained correctly; therefore, an adaptive compensation strategy is constructed based on the approximation capabilities of neural networks to solve the negative impact of sensor failures. The reinforcement-learning-based backstepping method is proposed to realize the optimal control of the system. Utilizing Lyapunov stability theory, it is shown that the designed controller enables the consensus error to converge to the prescribed performance bounds in fixed time and that all signals in the closed-loop system are bounded in probability. Finally, the simulation results prove the effectiveness of the proposed method.

## 1. Introduction

At the present time, multi-agent systems (MASs) have attracted widespread attention for their immense contributions to distributed coordination [1,2,3,4,5]. For example, a novel differential privacy bipartite consensus algorithm was proposed by Ma et al. [6], enabling consensus control in cooperative–competitive MASs. Wang et al. [7] designed the fixed-time formation controller for uncertain nonlinear MASs with time-varying actuator faults and random perturbations. To handle denial-of-service attacks and actuator faults in heterogeneous linear MASs, Zhang et al. [8] developed a resilient practical leader–follower consensus controller. Compared with the linear systems or ordinary nonlinear systems discussed earlier, stochastic nonlinear systems can be more widely applied in many practical engineering designs. Therefore, investigating stochastic nonlinear systems has greater practical significance and application value [9,10,11]. For instance, Zhu et al. [12] utilized the Nussbaum technique to resolve the issue of asymptotic consensus control in SNMASs. A compensator-based distributed controller for consensus control in SNMASs was developed by Li et al. [13]. However, all of the above studies ignored the effect of sensor faults.

In practical application, sensors often have unknown faults, which may lead to the failure of traditional control methods [14,15,16,17]. Therefore, compensating for sensor faults in MASs is crucial, as it enhances their security and reliability. For instance, Wu et al. [18] tackled sensor faults by designing resilient adaptive updates and state observers. To address sensor faults, Yu et al. [19] proposed an algorithm based on weighted average consensus and the unscented information filter.

As one of the important indicators to measure the performance of controllers, the problem of convergence time has been widely studied in recent years [20,21,22,23]. Although finite-time controls allow systems to converge quickly, their settling time depends on the initial value [24]. However, in many real-world scenarios, the initial values cannot be obtained in advance, so there is no control over the convergence time. Fixed-time control (FTC) methods have been proposed to solve the problem, where the convergence time can be predetermined and remains unaffected by the initial value [25,26,27]. To achieve the desired performance of the consensus error, the fixed-time prescribed performance control (FTPPC) method was designed [28,29,30,31]. To illustrate, Long et al. [32] adopted the performance strategy specified in a fixed-time frame to construct the control method, and realized the consensus control for nonlinear MASs under full state constraints.

To alleviate the challenge of directly solving the Hamilton–Jacobi–Bellman (HJB) equation when designing a consensus controller for MASs, a backstepping control design method utilizing reinforcement learning (RL) was ntroduced [33,34,35]. Wang et al. [36] used an actor–critic NN-based N-step backstepping structure to achieve consensus control for MASs. In [37,38], the idea of RL-based optimal backstepping control design was applied to tackle tracking control issues in ship systems and nonlinear systems with unknown dynamics, respectively. The above research has greatly pushed forward the development of nonlinear MAS consensus control.

Based on the above results, an improved FTPPC framework is proposed. To address the effects of sensor faults, an adaptive compensator based on neural networks (NNs) is constructed. Furthermore, the actor–critic structure within the reinforcement learning framework is utilized to develop an optimal backstepping method, ensuring that the consensus error remains within the FTPPC requirements, thereby achieving robust and reliable control.

(1) This paper presents an improved fixed-time prescribed performance framework. By constructing coordinate transformations, the consensus error can converge to a prescribed performance boundary in fixed time. Moreover, this framework overcomes the defect in finite-time control [20], where convergence time depends on the initial state.
(2) Considering the potential impact of sensor faults in real-world systems, which leads to a poor control performance, this paper utilizes neural networks to construct a sensor fault compensation mechanism. This enables the consensus error to efficiently converge to the prescribed performance boundary, even in the presence of unknown sensor faults.
(3) Compared with traditional backstepping methods [39], which do not account for system resource consumption, this paper uses RL to design an optimal control strategy, reducing the resource consumption associated with backstepping. Furthermore, compared with existing RL strategies [40], our approach uses a simpler adaptive laws form, ensuring that the RL network can be trained sufficiently and efficiently.

## 2. Preliminaries and Description

### 2.1. Graph Theory

Consider the digraph G=(V,E,A), representing the communication topology of MASs, where $V=\{v_{1},v_{2},\ldots ,v_{N}\}$ is the set of nodes consisting of *N* agents. E⊆V×V, $A=\left[a_{i,k}\right]\subseteq R^{N\times N}$ is the edges set and weighted adjacency matrix of the graph G, respectively. When $v_{i}$ can obtain information from $v_{k}$, denoted by $(v_{i},v_{k})\in E$, the weight $a_{i,k}\neq 0$ [41]. Otherwise, if there is no information exchange between $v_{i}$ and $v_{k}$, then $a_{i,k}=0$. In a directed graph G, the set of neighbors of $v_{i}$, denoted by $N_{i}$, is defined as all nodes connected to $v_{i}$. Specifically, $N_{i}=\left\{j|\left(v_{i},v_{j}\right)\in E\right\}$. The adjacency matrix *A* describes the connectivity between nodes in the graph, where $a_{i,j}$ represents the weight of the edge from $v_{j}$ to $v_{i}$. The degree matrix D is the sum of the weights of the edges adjacent to $v_{i}$ with $d_{i}=\sum a_{i,j}$. The Laplacian matrix L=D−A.

### 2.2. Neural Networks (NNs)

NNs have excellent function approximation and adaptive learning capabilities [42]. Based on the approximation properties of NNs, any continuous function $f\left(x\right):R^{n}\to R^{m}$ can be approximated by NNs as follows:

(1) $$f\left(x\right)=Y{*}^{T}\phi \left(x\right)+\varepsilon \left(x\right),$$

where $\phi \left(x\right)={\left[\phi_{1}\left(x\right),\ldots ,\phi_{p}\left(x\right)\right]}^{T}\in R^{p}$ with $\phi_{i}\left(x\right)=\exp\left(-{\left(x-\mu_{i}\right)}^{T}\left(x-\mu_{i}\right)/\sigma_{i}^{2}\right)\in R$, where $\sigma_{i}\in R$ and $\mu_{i}={\left[\mu_{i1},\ldots ,\mu_{in}\right]}^{T}\in R^{n}$ are the width and center of the Gaussian function, respectively. The approximation error $\varepsilon \left(x\right)\in R^{m}$ satisfies $∥\varepsilon \left(x\right)∥\leq \varepsilon^{*}$ with a positive constant $\varepsilon^{*}$. $Y^{*}\in R^{p}$ is the ideal weight vector, which satisfies the following:

(2) $$Y^{*}:=\arg\min_{Y\in R^{p}}\left\{\underset{x\in \Omega_{x}}{\sup}∥f\left(x\right)-Y^{T}\phi \left(x\right)∥\right\}.$$

### 2.3. System Description

Consider the following SNMASs with sensor faults:

(3) $$\left\{\begin{array}{cc} dx_{i,j}= & \left[f_{i,j}\left({\bar{x}}_{i,j}\right)+x_{i,j+1}\right]dt+g_{i,j}\left({\bar{x}}_{i,j}\right)d\omega \\ dx_{i,n}= & \left[f_{i,n}\left({\bar{x}}_{i,n}\right)+u_{i}\right]dt+g_{i,n}\left({\bar{x}}_{i,n}\right)d\omega \\ y_{i}^{f}= & \varrho_{i}x_{i,1}+\rho_{i},1\leq i\leq N,1\leq j\leq n-1, \end{array}\right.$$

where $f_{i,j}\left(\cdot \right):R^{n}\to R^{n}$ and $g_{i,j}\left(\cdot \right):R^{n}\to R^{n\times r}$, ω is *r*-dimensional independent standard wiener process, $x_{i,j}$ is state, ${\bar{x}}_{i,j}={\left[x_{i,1},\ldots ,x_{i,j}\right]}^{T}\in R^{j}$ is the system state vector, $u_{i}\in R$ is the control input, and $y_{i}^{f}\in R$ is the untrue state measured by the sensor. $\varrho_{i}$ and $\rho_{i}$ are the sensor fault parameters, which will be defined later.

**Definition** **1** (See [43])**.** *A sensor fault occurs if the output $y_{i}^{f}\left(t\right)$ of the sensor measuring the system output signal $x_{i,1}\left(t\right)\in R$ satisfies $y_{i}^{f}\left(t\right)=\varrho_{i}x_{i,1}\left(t\right)+\rho_{i}$, where the unknown parameters $\varrho_{i}$ and $\rho_{i}$ satisfy ${\bar{\varrho }}_{i,\min}\leq \varrho_{i}\leq 1$ and $-{\bar{\rho }}_{i}\leq \rho_{i}\leq {\bar{\rho }}_{i}$, respectively. ${\bar{\varrho }}_{i,\min}$ and ${\bar{\rho }}_{i}$ are constants.*

**Definition** **2** (See [21])**.** *Consider the following stochastic nonlinear system:*

(4) dx=f(x)dt+g(x)dω,

*For the system (4) and any twice-differentiable function V(x), based on Itô’s Lemma and the stochastic differentials properties ${\left(d\omega \right)}^{2}=dt$, define the differential operator L and derivative d as follows:*

(5) $$LV=\frac{\partial V}{\partial x}f\left(x\right)+\frac{1}{2}Tr\left\{g^{T}\left(x\right)\frac{\partial^{2}V}{\partial x^{2}}g\left(x\right)\right\},$$

(6) $$dV=LVdt+\frac{\partial V}{\partial x}gd\omega ,$$

*where Tr{A} is the trace of matrix A.*

**Assumption** **1.** *At least one follower can directly receive information from the leader.*

**Lemma** **1** (See [44])**.** *For any m,n,λ>0,κ,ν∈R and (m−1)(n−1)=1, we have the following:*

(7) $$\kappa^{T}\nu \leq \frac{\lambda^{m}}{m}{∥\kappa ∥}^{m}+\frac{1}{n\lambda^{n}}{∥\nu ∥}^{n}.$$

For clarity, a list of key variables and a list of abbreviations used in this paper are provided in Appendix A.

## 3. Adaptive Optimal Consensus Controller Design and Stability Analysis

The backstepping technique is employed for controller design.

### 3.1. Adaptive Optimal Consensus Controller Design

For agent *i* in SNMASs (3), let $f_{si}=\left(\varrho_{i}-1\right)x_{i,1}+\rho_{i}$, we have

(8) $$y_{i}^{f}\left(t\right)=x_{i,1}+f_{si}.$$

The consensus error is defined as

(9) $$s_{i}\left(t\right)=\sum_{k\in N_{i}}a_{i,k}\left(y_{i}^{f}-y_{k}^{f}\right)+b_{i}\left(y_{i}^{f}-y_{r}\left(t\right)\right),$$

where $y_{r}\left(t\right)$ is a reference signal. $b_{i}=1$ means that the leader information can be received by agent *i*; otherwise, $b_{i}=0$.

To achieve the FTPPC requirement for $s_{i}\left(t\right)$, this paper chooses the fixed-time performance function (FTPF) as follows:

(10) $$h\left(t\right)=\left\{\begin{array}{cccc} (h_{0}- & h_{\tilde{T}}){\left(1-\frac{\lambda t}{\iota }\right)}^{\iota }+h_{\tilde{T}} & , & 0\leq t<\tilde{T}\\ & h_{\tilde{T}} & , & t\geq \tilde{T}, \end{array}\right.$$

where ι≥n and λ>0 are the design parameters, $h_{0}=h\left(0\right)>h_{\tilde{T}}=lim_{t\to \tilde{T}}h\left(t\right)>0$, and the settling time $\tilde{T}=\iota /\lambda <\infty$.

Achieving the asymmetric FTPPC requires ensuring that the following inequalities hold:

(11) $$-\delta_{m}h\left(t\right)<s_{i}\left(t\right)<\delta_{M}h\left(t\right),t>0.$$

where $\delta_{m}$ and $\delta_{M}$ are positive asymmetric design parameters.

Then, we make

(12) $$\begin{array}{cc} \eta_{i}\left(\mu_{i}\right) & =\left(\delta_{M}e^{\mu_{i}}-\delta_{m}e^{-\mu_{i}}\right)/\left(e^{\mu_{i}}+e^{-\mu_{i}}\right),\\ s_{i}\left(t\right) & =h\left(t\right)\eta_{i}\left(\mu_{i}\right). \end{array}$$

Then, one has

$$\mu_{i}\left(t\right)=\eta_{i}^{-1}\left(\frac{s_{i}\left(t\right)}{h(t)}\right)=\frac{1}{2}\ln\frac{\eta_{i}+\delta_{m}}{\delta_{M}-\eta_{i}}.$$

For convenience of description, we let

$$\begin{array}{cc} \varphi_{i}= & \left(1/2h\right)(\left[1/\left(\eta_{i}+\delta_{m}\right)\right]-\left[1/\left(\eta_{i}-\delta_{M}\right)\right]),\\ \psi_{i}= & -\left(1/2h\right)(\left[1/\left(\eta_{i}+\delta_{m}\right)\right]+\left[1/\left(\eta_{i}-\delta_{M}\right)\right]). \end{array}$$

Then, avoiding the zero equilibrium point issue, we propose the following state coordinate transformation for the *n*-step backstepping method.

(13) $$\left\{\begin{array}{cc} z_{i,1} & =\mu_{i}-\frac{1}{2}\ln\frac{\delta_{m}}{\delta_{M}}\\ z_{i,j} & =x_{i,j}-{\hat{\alpha }}_{i,j-1},j=2,\ldots ,n, \end{array}\right.$$

where ${\hat{\alpha }}_{i,j-1}$ is the approximate ideal virtual control.

**Step 1:** From (13), the derivative of $z_{i,1}$ is obtained as

(14) $$\begin{array}{cc} dz_{i,1}= & \varphi_{i}(\left(d_{i}+b_{i}\right)x_{i,2}+F_{si}+F_{pi}\\ & -\beta_{i,1}+\Gamma_{i,1})dt+{℧}_{i,1}d\omega , \end{array}$$

where $F_{si}=\left(d_{i}+b_{i}\right)f_{i,1}\left(x_{i,1}\right)-\sum_{k\in N_{i}}a_{i,k}f_{k,1}\left(x_{k,1}\right),\Gamma_{i,1}={\left(d_{i}+b_{i}\right)}^{2}\varrho_{i}^{2}\psi_{i}g_{i,1}^{T}g_{i,1}+d_{k}^{2}\varrho_{k}^{2}\psi_{i}g_{k,1}^{T}g_{k,1}$ $-\sum_{k\in N_{i}}a_{i,k}x_{k,2},F_{pi}=\left(d_{i}+b_{i}\right)f_{pi}-\sum_{k\in N_{i}}a_{i,k}f_{pk},\beta_{i,1}=\frac{\dot{h}s_{i}\left(t\right)}{h}+b_{i}\dot{y_{r}}$ and ${℧}_{i,1}=\varphi_{i}\left(d_{i}+b_{i}\right)\varrho_{i}g_{i,1}+\varphi_{i}b_{k}\varrho_{k}g_{k,1}$,$f_{pi}={\dot{f}}_{si},f_{pk}={\dot{f}}_{sk}$.

The unknown smooth functions $F_{si}$ and $F_{pi}$ are approximated by NNs as

(15) $$\begin{array}{cc} F_{pi} & =Y_{pi}^{*T}\phi_{pi}\left({\bar{x}}_{i,2},{\bar{x}}_{k,2}\right)+\varepsilon_{pi}\left({\bar{x}}_{i,2},{\bar{x}}_{k,2}\right),\\ F_{si} & =Y_{si}^{*T}\phi_{si}\left({\bar{x}}_{i,2}\right)+\varepsilon_{si}\left({\bar{x}}_{i,2}\right), \end{array}$$

where ${\bar{x}}_{k,2}={\left[x_{k,1},x_{k,2}\right]}^{T}$, $\varepsilon_{si}\left({\bar{x}}_{i,2}\right)$ and $\varepsilon_{pi}\left({\bar{x}}_{i,2},{\bar{x}}_{k,2}\right)$ are defined as the approximation errors.

The value function is designed as

(16) $$J_{i,1}\left(z_{i,1}\left(t\right)\right)=\lim_{t_{f}\to \infty }\frac{1}{t_{f}}\int_{t}^{t_{f}}W_{i,1}d\tau ,$$

where $W_{i,1}=z_{i,1}^{4}+\alpha_{i,1}^{2}+\sum_{k\in N_{i}}\alpha_{k,1}^{2}$, $t_{f}$ is the control end time.

Then, define an optimal value function as

(17) $$\begin{array}{cc} J_{i,1}^{*}\left(z_{i,1}\right) & =\min_{\alpha_{i,1}\in \psi_{i}\left(\Omega_{i,1}\right)}\left[\lim_{t_{f}\to \infty }\frac{1}{t_{f}}\int_{t}^{t_{f}}W_{i,1}d\tau \right]=\lim_{t_{f}\to \infty }\frac{1}{t_{f}}\int_{t}^{t_{f}}W_{i,1}^{*}d\tau , \end{array}$$

where $\Omega_{i,1}$ is the compact set. $W_{i,1}^{*}=z_{i,1}^{4}+\alpha_{i,1}^{*2}+\sum_{k\in N_{i}}\alpha_{k,1}^{*2}$. $\psi_{i}\left(\Omega_{i,1}\right)$ is the available control set. $\alpha_{i,1}^{*}$ is the ideal virtual control.

Define $V_{i,1}\left(z_{i,1}\left(t\right)\right)=\int_{t}^{t_{f}}W_{i,1}d\tau$, thus, $V_{i,1}^{*}\left(z_{i,1}\left(t\right)\right)=\int_{t}^{t_{f}}W_{i,1}^{*}d\tau$, one has

(18) $$\begin{array}{cc} J_{i,1}^{*}\left(z_{i,1}\right)= & \lim_{t_{f}\to \infty }\frac{1}{t_{f}}\int_{t}^{t+\Delta t}W_{i,1}^{*}d\tau +\lim_{t_{f}\to \infty }\frac{1}{t_{f}}V_{i,1}^{*}\left(z_{i,1}\left(t+\Delta t\right)\right). \end{array}$$

Then, one has

(19) $$\begin{array}{cc} \lim_{t_{f}\to \infty }\frac{1}{t_{f}}[ & \lim_{\Delta t\to 0}\frac{1}{\Delta t}\int_{t}^{t+\Delta t}W_{i,1}^{*}d\tau +\lim_{\Delta t\to 0}\frac{V_{i,1}^{*}\left(z_{i,1}\left(t+\Delta t\right)\right)-V_{i,1}^{*}\left(z_{i,1}\left(t\right)\right)}{\Delta t}]=0. \end{array}$$

It means that

(20) $$\begin{array}{c} \lim_{t_{f}\to \infty }\frac{1}{t_{f}}\left[W_{i,1}^{*}+\frac{\partial V_{i,1}^{*}\left(z_{i,1}\right)}{\partial t}\right]=0. \end{array}$$

Based on (5) and (6) and $x_{i,2}$ is regarded as $\alpha_{i,1}^{*}$, one has

(21) $$\begin{array}{cc} \frac{\partial V_{i,1}^{*}\left(z_{i,1}\right)}{\partial t}= & \frac{\partial V_{i,1}^{*}\left(z_{i,1}\right)}{\partial z_{i,1}}\varphi_{i}\left(\left(d_{i}+b_{i}\right)\alpha_{i,1}^{*}+F_{si}+F_{pi}-\beta_{i,1}+\Gamma_{i,1}\right)+\frac{1}{2}\frac{\partial^{2}V_{i,1}^{*}\left(z_{i,1}\right)}{\partial z_{i,1}^{2}}{∥{℧}_{i,1}∥}^{2}. \end{array}$$

Now, we have the HJB equation as follows:

(22) $$\begin{array}{c} H_{i,1}\left(z_{i,1},\alpha_{i,1}^{*},\alpha_{k,1}^{*},\frac{\partial V_{i,1}^{*}\left(z_{i,1}\right)}{\partial z_{i,1}}\right)=W_{i,1}^{*}+\frac{\partial V_{i,1}^{*}\left(z_{i,1}\right)}{\partial t}=0. \end{array}$$

By solving the equation $\partial H_{i,1}/\partial \alpha_{i,1}^{*}=0$, one has

(23) $$\begin{array}{c} \alpha_{i,1}^{*}=-\frac{\varphi_{i}\left(d_{i}+b_{i}\right)}{2}\frac{\partial V_{i,1}^{*}\left(z_{i,1}\right)}{\partial z_{i,1}}. \end{array}$$

Then, we let

(24) $$\begin{array}{cc} \frac{\partial V_{i,1}^{*}\left(z_{i,1}\right)}{\partial z_{i,1}}= & \frac{1}{\varphi_{i}{\left(d_{i}+b_{i}\right)}^{2}}\{2\eta_{i,1}\varphi_{i}z_{i,1}^{3}+\frac{2{\bar{\eta }}_{i,1}}{\varphi_{i}}z_{i,1}+\frac{\phi_{si}^{T}\phi_{si}}{\pi^{2}}z_{i,1}^{3}\varphi_{i}\Theta_{si,1}^{*}\\ & -2\beta_{i,1}+V_{i,1}^{c}\left(z_{i,1}\right)+\frac{\phi_{pi}^{T}\phi_{pi}}{\pi^{2}}z_{i,1}^{3}\varphi_{i}\Theta_{pi,1}^{*}+\frac{\phi_{fi,1}^{T}\phi_{fi,1}}{\pi^{2}}z_{i,1}^{3}\varphi_{i}\Theta_{i,1}^{*}\}, \end{array}$$

where $\eta_{i,1},{\bar{\eta }}_{i,1}$ and π are design positive constants. $\Theta_{i,1}^{*},\Theta_{si,1}^{*},\Theta_{pi,1}^{*}$ are the ideal weights, which will be defined later. $V_{i,1}^{c}\left(z_{i,1}\right)=-2\eta_{i,1}\varphi_{i}z_{i,1}^{3}-\frac{\phi_{fi,1}^{T}\phi_{fi,1}}{2\pi^{2}}z_{i,1}^{3}\varphi_{i}\Theta_{i,1}^{*}-\frac{2{\bar{\eta }}_{i,1}}{\varphi_{i}}z_{i,1}+\frac{2\dot{h}s_{i}\left(t\right)}{h}-\frac{\phi_{si}^{T}\phi_{si}}{2\pi^{2}}z_{i,1}^{3}\varphi_{i}\Theta_{si,1}^{*}-\frac{\phi_{pi}^{T}\phi_{pi}}{2\pi^{2}}z_{i,1}^{3}\varphi_{i}\Theta_{pi,1}^{*}+\varphi_{i}{\left(d_{i}+b_{i}\right)}^{2}\frac{\partial V_{i,1}^{*}\left(z_{i,1}\right)}{\partial z_{i,1}}$.

From (23) and (24), rewrite (23) as

(25) $$\begin{array}{cc} \alpha_{i,1}^{*}= & -\frac{1}{d_{i}+b_{i}}\{\eta_{i,1}\varphi_{i}z_{i,1}^{3}+\frac{{\bar{\eta }}_{i,1}}{\varphi_{i}}z_{i,1}+\frac{\phi_{fi,1}^{T}\phi_{fi,1}}{2\pi^{2}}z_{i,1}^{3}\varphi_{i}\Theta_{i,1}^{*}\\ & +\frac{1}{2}V_{i,1}^{c}\left(z_{i,1}\right)+\frac{\phi_{si}^{T}\phi_{si}}{2\pi^{2}}z_{i,1}^{3}\varphi_{i}\Theta_{si,1}^{*}+\frac{\phi_{pi}^{T}\phi_{pi}}{2\pi^{2}}z_{i,1}^{3}\varphi_{i}\Theta_{pi,1}^{*}-\beta_{i,1}\}. \end{array}$$

$V_{i,1}^{c}\left(z_{i,1}\right)$ is approximated by NNs as

(26) $$\begin{array}{c} V_{i,1}^{c}\left(z_{i,1}\right)=Y_{i,1}^{{*}^{T}}\phi_{i,1}\left(z_{i,1}\right)+\varepsilon_{i,1}\left(z_{i,1}\right), \end{array}$$

where $∥\varepsilon_{i,1}\left(z_{i,1}\right)∥\leq \varepsilon_{i,1}^{*}$ with constant $\varepsilon_{i,1}^{*}>0$.

Thus, from (25) and (26), as ideal weights $\Theta_{i,1}^{*},\Theta_{si,1}^{*}$, $\Theta_{pi,1}^{*}$ and $Y_{i,1}^{*}$ are unknown parameters, thus, using ${\hat{\Theta }}_{i,1},{\hat{\Theta }}_{si,1}$, ${\hat{\Theta }}_{pi,1},{\hat{Y}}_{i,1}$ to estimate $\Theta_{i,1}^{*},\Theta_{si,1}^{*},\Theta_{pi,1}^{*},Y_{i,1}^{*}$, respectively.

To optimize the control performance, an RL method with an actor–critic structure is proposed as follows:

(27) $$\begin{array}{cc} \frac{\partial {\hat{V}}_{i,1}\left(z_{i,1}\right)}{\partial z_{i,1}}= & \frac{1}{\varphi_{i}{\left(d_{i}+b_{i}\right)}^{2}}\{2\eta_{i,1}\varphi_{i}z_{i,1}^{3}+\frac{2{\bar{\eta }}_{i,1}}{\varphi_{i}}z_{i,1}-2\beta_{i,1}+\frac{\phi_{fi,1}^{T}\phi_{fi,1}}{\pi^{2}}z_{i,1}^{3}\varphi_{i}{\hat{\Theta }}_{i,1}\\ & +{\hat{Y}}_{i,c1}\phi_{i,1}\left(z_{i,1}\right)+\frac{\phi_{si}^{T}\phi_{si}}{\pi^{2}}z_{i,1}^{3}\varphi_{i}{\hat{\Theta }}_{si,1}+\frac{\phi_{pi}^{T}\phi_{pi}}{\pi^{2}}z_{i,1}^{3}\varphi_{i}{\hat{\Theta }}_{pi,1}\}, \end{array}$$

(28) $$\begin{array}{cc} {\hat{\alpha }}_{i,1}=-\frac{1}{d_{i}+b_{i}} & \{\eta_{i,1}\varphi_{i}z_{i,1}^{3}+\frac{\phi_{fi,1}^{T}\phi_{fi,1}}{2\pi^{2}}z_{i,1}^{3}\varphi_{i}{\hat{\Theta }}_{i,1}+\frac{\phi_{si}^{T}\phi_{si}}{2\pi^{2}}z_{i,1}^{3}\varphi_{i}{\hat{\Theta }}_{si,1}\\ & +\frac{{\bar{\eta }}_{i,1}}{\varphi_{i}}z_{i,1}-\beta_{i,1}+\frac{\phi_{pi}^{T}\phi_{pi}}{2\pi^{2}}z_{i,1}^{3}\varphi_{i}{\hat{\Theta }}_{pi,1}+\frac{1}{2}{\hat{Y}}_{i,a1}\phi_{i,1}\left(z_{i,1}\right)\}, \end{array}$$

where ${\hat{Y}}_{i,a1}$ and ${\hat{Y}}_{i,c1}$ estimate $Y_{i,1}^{*}$, $\frac{\partial {\hat{V}}_{i,1}\left(z_{i,1}\right)}{\partial z_{i,1}}$ estimates $\frac{\partial V_{i,1}^{*}\left(z_{i,1}\right)}{\partial z_{i,1}}$.

**Remark** **1.** *Solving the HJB equation analytically is challenging [45], so we employ a reinforcement learning approach to approximate its solution. Specifically, we construct an actor-critic network architecture: the actor interacts with the environment to optimize the policy based on the feedback, while the critic evaluates the current policy to improve the value function. Through the interaction between the actor and critic networks, an approximate solution to the HJB equation is obtained, thereby achieving optimal control.*

Thus, from (22), (27) and (28), the approximation HJB function is

(29) $$\begin{array}{cc} H_{i,1}\left(z_{i,1},{\hat{\alpha }}_{i,1},{\hat{\alpha }}_{k,1},\frac{\partial {\hat{V}}_{i,1}\left(z_{i,1}\right)}{\partial z_{i,1}}\right)= & z_{i,1}^{4}+{\hat{\alpha }}_{i,1}^{2}+\sum_{k\in N_{i}}{\hat{\alpha }}_{k,1}^{2}+\frac{\partial {\hat{V}}_{i,1}\left(z_{i,1}\right)}{\partial z_{i,1}}\varphi_{i}(\left(d_{i}+b_{i}\right){\hat{\alpha }}_{i,1}\\ & +F_{si}+F_{pi}-\beta_{i,1}+\Gamma_{i,1})+\frac{1}{2}\frac{\partial^{2}{\hat{V}}_{i,1}\left(z_{i,1}\right)}{\partial z_{i,1}^{2}}{∥{℧}_{i,1}∥}^{2}. \end{array}$$

Define the Bellman error as

(30) $$\begin{array}{cc} \epsilon_{i,1}= & H_{i,1}\left(z_{i,1},{\hat{\alpha }}_{i,1},{\hat{\alpha }}_{k,1},\frac{\partial {\hat{V}}_{i,1}\left(z_{i,1}\right)}{\partial z_{i,1}}\right)-H_{i,1}\left(z_{i,1},\alpha_{i,1}^{*},\alpha_{k,1}^{*},\frac{\partial V_{i,1}^{*}\left(z_{i,1}\right)}{\partial z_{i,1}}\right)\\ = & H_{i,1}\left(z_{i,1},{\hat{\alpha }}_{i,1},{\hat{\alpha }}_{k,1},\frac{\partial {\hat{V}}_{i,1}\left(z_{i,1}\right)}{\partial z_{i,1}}\right). \end{array}$$

${\hat{\alpha }}_{i,1}$ is expected as a unique solution to make $\epsilon_{i,1}\to 0$. If $\epsilon_{i,1}=0$ is held and has unique solution, it is equivalent to the following:

(31) $$\begin{array}{c} \frac{\partial \epsilon_{i,1}}{\partial {\hat{Y}}_{i,a1}}=\frac{\phi_{i,1}\left(z_{i,1}\right)\phi_{i,1}^{T}\left(z_{i,1}\right)}{2{\left(d_{i}+b_{i}\right)}^{2}}\left({\hat{Y}}_{i,a1}-{\hat{Y}}_{i,c1}\right)=0_{N\times 1}. \end{array}$$

To formulate the actor updating law for training the weight as given in (31), we design a positive definite function P(t) as follows:

(32) $$\begin{array}{cc} P(t)= & \frac{1}{2{\left(d_{i}+b_{i}\right)}^{2}}{\left({\hat{Y}}_{i,a1}-{\hat{Y}}_{i,c1}\right)}^{T}\varpi_{i,1}\left({\hat{Y}}_{i,a1}-{\hat{Y}}_{i,c1}\right), \end{array}$$

where $\varpi_{i,1}=\phi_{i,1}\left(z_{i,1}\right)\phi_{i,1}^{T}\left(z_{i,1}\right)+j_{i,1}I$, $j_{i,1}>0$ is the design constant and $I\in R^{p\times p}$ is a identity matrix.

Obviously, P(t)=0 is synonymous with (31). Consequently, the adaptive updating law ${\dot{\hat{Y}}}_{i,a1}$ is derived from the negative gradient of P(t).

(33) $$\begin{array}{cc} {\dot{\hat{Y}}}_{i,a1} & =-\gamma_{i,a1}\frac{\partial P(t)}{\partial {\hat{Y}}_{i,a1}}=-\frac{\gamma_{i,a1}}{{\left(d_{i}+b_{i}\right)}^{2}}\varpi_{i,1}\left({\hat{Y}}_{i,a1}-{\hat{Y}}_{i,c1}\right). \end{array}$$

**Remark** **2.** *The actor updating law in [33] was constructed as ${\dot{\hat{Y}}}_{i,a1}=-\gamma_{i,a1}\phi_{i,1}\phi_{i,1}^{T}\left({\hat{Y}}_{i,a1}-{\hat{Y}}_{i,c1}\right)$. When ${\hat{Y}}_{i,a1}-{\hat{Y}}_{i,c1}$ falls on the zero feature vector of $\phi_{i,1}\phi_{i,1}^{T}$, the training will be terminated prematurely. Therefore, $j_{i,1}I$ is introduced in (33) for sufficient training.*

Design adaptive law ${\dot{\hat{W}}}_{i,c1}$ of critic NNs, and adaptive law ${\dot{\hat{\Theta }}}_{i,1},{\dot{\hat{\Theta }}}_{si,1}$ and ${\dot{\hat{\Theta }}}_{pi,1}$ as

(34) $$\begin{array}{cc} {\dot{\hat{Y}}}_{i,c1}= & -\frac{\gamma_{i,c1}}{{\left(d_{i}+b_{i}\right)}^{2}}\varpi_{i,1}{\hat{Y}}_{i,c1}-\frac{\varphi_{i}}{2}\phi_{i,1}\left(z_{i,1}\right)z_{i,1}^{3}, \end{array}$$

(35) $$\begin{array}{c} {\dot{\hat{\Theta }}}_{i,1}=z_{i,1}^{6}\varphi_{i}^{2}\frac{\gamma_{i,1}}{2\pi^{2}}\phi_{fi,1}^{T}\phi_{fi,1}-\sigma_{i,1}{\hat{\Theta }}_{i,1}, \end{array}$$

(36) $$\begin{array}{c} {\dot{\hat{\Theta }}}_{si,1}=z_{i,1}^{6}\varphi_{i}^{2}\frac{{\bar{\gamma }}_{i,1}}{2\pi^{2}}\phi_{si}^{T}\phi_{si}-\sigma_{si,1}{\hat{\Theta }}_{si,1}, \end{array}$$

(37) $$\begin{array}{c} {\dot{\hat{\Theta }}}_{pi,1}=z_{i,1}^{6}\varphi_{i}^{2}\frac{{\underline{\gamma }}_{i,1}}{2\pi^{2}}\phi_{pi}^{T}\phi_{pi}-\sigma_{pi,1}{\hat{\Theta }}_{pi,1}, \end{array}$$

where $\gamma_{i,a1}>0$ and $\gamma_{i,c1}>0$ are learning rates, and $\gamma_{i,a1}>\gamma_{i,c1}$. $\gamma_{i,1},{\bar{\gamma }}_{i,1},{\underline{\gamma }}_{i,1},\sigma_{i,1},\sigma_{si,1}$ and $\sigma_{pi,1}$ are positive constants.

**Step j (2≤j≤n−1):** From (3) and (13), similar to (14) one has

(38) $$\begin{array}{cc} dz_{i,j}= & \left(x_{i,j+1}-\beta_{i,j}-\Gamma_{i,j}-\Phi_{i,j}\right)dt+{℧}_{i,j}d\omega , \end{array}$$

where $\beta_{i,j}=\sum_{m=1}^{j-1}\frac{\partial {\hat{\alpha }}_{i,j-1}}{\partial {\hat{Y}}_{i,am}}{\dot{\hat{Y}}}_{i,am}+\sum_{m=1}^{j-1}\frac{\partial {\hat{\alpha }}_{i,j-1}}{\partial {\hat{\Theta }}_{i,m}}{\dot{\hat{\Theta }}}_{i,m}+\sum_{m=1}^{j-1}\frac{\partial {\hat{\alpha }}_{i,j-1}}{\partial {\hat{\Theta }}_{si,m}}{\dot{\hat{\Theta }}}_{si,m}+\sum_{m=1}^{j-1}\frac{\partial {\hat{\alpha }}_{i,j-1}}{\partial {\hat{\Theta }}_{pi,m}}{\dot{\hat{\Theta }}}_{pi,m}-\frac{\partial {\hat{\alpha }}_{i,j-1}}{\partial z_{i,1}}\varphi_{i}\beta_{i,1},\Gamma_{i,j}=\sum_{m=1}^{j-1}\frac{\partial {\hat{\alpha }}_{i,j-1}}{\partial x_{i,m}}x_{i,m+1}-f_{i,j}+\;\frac{1}{2}\left(\frac{\partial^{2}{\hat{\alpha }}_{i,j-1}}{\partial z_{i,1}^{2}}\varphi_{i}^{2}b_{i}^{2}\varrho_{i}^{2}g_{i,1}^{T}g_{i,1}+\sum_{p,q=1}^{j-1}\frac{\partial^{2}{\hat{\alpha }}_{i,j-1}}{\partial x_{i,p}\partial x_{i,q}}g_{i,p}^{T}g_{i,q}+2\sum_{m=1}^{j-1}\frac{\partial^{2}{\hat{\alpha }}_{i,j-1}}{\partial z_{i,1}\partial x_{i,m}}\varphi_{i}b_{i}\varrho_{i}g_{i,m}^{T}g_{i,1}\right)+\frac{\partial {\hat{\alpha }}_{i,j-1}}{\partial z_{i,1}}\varphi_{i}\Gamma_{i,1}+\frac{\partial {\hat{\alpha }}_{i,j-1}}{\partial z_{i,1}}\varphi_{i}F_{si}+\frac{\partial {\hat{\alpha }}_{i,j-1}}{\partial z_{i,1}}\varphi_{i}F_{pi}+\sum_{m=1}^{j-1}\frac{\partial {\hat{\alpha }}_{i,j-1}}{\partial x_{i,m}}f_{i,m},$ $\Phi_{i,j}=\frac{\partial {\hat{\alpha }}_{i,j-1}}{\partial z_{i,1}}\varphi_{i}\left(d_{i}+b_{i}\right)x_{i,j}$ and ${℧}_{i,j}=g_{i,j}-\sum_{m=1}^{j-1}\frac{\partial {\hat{\alpha }}_{i,j-1}}{\partial x_{i,m}}g_{i,m}-\frac{\partial {\hat{\alpha }}_{i,j-1}}{\partial z_{i,1}}{℧}_{i,1}$.

Similar to (16) and (17), define an optimal value function as follows:

(39) $$\begin{array}{cc} J_{i,j}^{*}\left(z_{i,j}\right) & =\lim_{t_{f}\to \infty }\frac{1}{t_{f}}\int_{t}^{t_{f}}W_{i,j}^{*}d\tau , \end{array}$$

where $W_{i,j}^{*}=z_{i,j}^{4}+\alpha_{i,j}^{*2}$.

Then, similar to (18)–(24), one has

(40) $$\begin{array}{cc} \frac{\partial V_{i,j}^{*}\left(z_{i,j}\right)}{\partial z_{i,j}}= & 2\left(\eta_{i,j}+\frac{1}{2}{\left(\frac{\partial {\hat{\alpha }}_{i,j-1}}{\partial z_{i,1}}\right)}^{2}\varphi_{i}^{2}\right)z_{i,j}^{3}+2\left({\bar{\eta }}_{i,j}+\delta^{2}+\frac{3}{4}{\bar{\varepsilon }}_{i,j}^{\frac{4}{3}}{\bar{\Phi }}_{i,j}\right)z_{i,j}\\ & +V_{i,j}^{c}-2\beta_{i,j}+\frac{\phi_{fi,j}^{T}\phi_{fi,j}}{\pi^{2}}z_{i,j}^{3}\Theta_{i,j}^{*}, \end{array}$$

(41) $$\begin{array}{cc} \alpha_{i,j}^{*}= & -\{\left(\eta_{i,j}+\frac{1}{2}{\left(\frac{\partial {\hat{\alpha }}_{i,j-1}}{\partial z_{i,1}}\right)}^{2}\varphi_{i}^{2}\right)z_{i,j}^{3}+\left({\bar{\eta }}_{i,j}+\delta^{2}+\frac{3}{4}{\bar{\varepsilon }}_{i,j}^{\frac{4}{3}}{\bar{\Phi }}_{i,j}\right)z_{i,j}\\ & +\frac{1}{2}V_{i,j}^{c}-\beta_{i,j}+\frac{\phi_{fi,j}^{T}\phi_{fi,j}}{2\pi^{2}}z_{i,j}^{3}\Theta_{i,j}^{*}\}, \end{array}$$

where $\eta_{i,j}>0,{\bar{\eta }}_{i,j}>0$, $\Theta_{i,j}^{*}$ is the ideal weight, which will be defined later. $V_{i,j}^{c}\left(z_{i,j}\right)=\frac{\partial V_{i,j}^{*}\left(z_{i,j}\right)}{\partial z_{i,j}}+2\beta_{i,j}-2\left(\eta_{i,j}+\frac{1}{2}{\left(\frac{\partial {\hat{\alpha }}_{i,j-1}}{\partial z_{i,1}}\right)}^{2}\varphi_{i}^{2}\right)z_{i,j}^{3}-2\left({\bar{\eta }}_{i,j}+\delta^{2}+\frac{3}{4}{\bar{\varepsilon }}_{i,j}^{\frac{4}{3}}{\bar{\Phi }}_{i,j}\right)z_{i,j}-\frac{\phi_{fi,j}^{T}\phi_{fi,j}}{\pi^{2}}z_{i,j}^{3}\Theta_{i,j}^{*}$.

Similar to (26)–(28), one has

(42) $$\begin{array}{cc} \frac{\partial {\hat{V}}_{i,j}\left(z_{i,j}\right)}{\partial z_{i,j}}= & 2\left(\eta_{i,j}+\frac{1}{2}{\left(\frac{\partial {\hat{\alpha }}_{i,j-1}}{\partial z_{i,1}}\right)}^{2}\varphi_{i}^{2}\right)z_{i,j}^{3}+2\left({\bar{\eta }}_{i,j}+\delta^{2}+\frac{3}{4}{\bar{\varepsilon }}_{i,j}^{\frac{4}{3}}{\bar{\Phi }}_{i,j}\right)z_{i,j}\\ & +{\hat{Y}}_{i,cj}^{T}\phi_{i,j}\left(z_{i,j}\right)+\frac{\phi_{fi,j}^{T}\phi_{fi,j}}{\pi^{2}}z_{i,j}^{3}{\hat{\Theta }}_{i,j}-2\beta_{i,j}, \end{array}$$

(43) $$\begin{array}{cc} {\hat{\alpha }}_{i,j}= & -\{\left(\eta_{i,j}+\frac{1}{2}{\left(\frac{\partial {\hat{\alpha }}_{i,j-1}}{\partial z_{i,1}}\right)}^{2}\varphi_{i}^{2}\right)z_{i,j}^{3}+({\bar{\eta }}_{i,j}+\frac{3}{4}{\bar{\varepsilon }}_{i,j}^{\frac{4}{3}}{\bar{\Phi }}_{i,j}\\ & +\delta^{2})z_{i,j}+\frac{1}{2}{\hat{Y}}_{i,aj}^{T}\phi_{i,j}\left(z_{i,j}\right)+\frac{\phi_{fi,j}^{T}\phi_{fi,j}}{2\pi^{2}}z_{i,j}^{3}{\hat{\Theta }}_{i,j}-\beta_{i,j}\}. \end{array}$$

Similar to (29)–(37), design the adaptive laws ${\dot{\hat{\Theta }}}_{i,j}$, ${\dot{\hat{Y}}}_{i,cj}$ and ${\dot{\hat{Y}}}_{i,aj}$ as

(44) $$\begin{array}{c} {\dot{\hat{\Theta }}}_{i,j}=z_{i,j}^{6}\frac{\gamma_{i,j}}{2\pi^{2}}\phi_{fi,j}^{T}\phi_{fi,j}-\sigma_{i,j}{\hat{\Theta }}_{i,j}, \end{array}$$

(45) $$\begin{array}{c} {\dot{\hat{Y}}}_{i,aj}=-\gamma_{i,aj}\varpi_{i,j}\left({\hat{Y}}_{i,aj}-{\hat{Y}}_{i,cj}\right), \end{array}$$

(46) $$\begin{array}{c} {\dot{\hat{Y}}}_{i,cj}=-\gamma_{i,cj}\varpi_{i,j}{\hat{Y}}_{i,cj}-\frac{1}{2}\phi_{i,j}\left(z_{i,j}\right)z_{i,j}^{3}, \end{array}$$

where $\varpi_{i,j}=\phi_{i,j}\left(z_{i,j}\right)\phi_{i,j}^{T}\left(z_{i,j}\right)+j_{i,j}I$, $j_{i,j}$, $\gamma_{i,j}$ and $\sigma_{i,j}$ are the design positive constants, $\gamma_{i,aj}>0$ and $\gamma_{i,cj}>0$ are learning rates, and $\gamma_{i,aj}>\gamma_{i,cj}$.

**Step n:** From (3) and (13), similar to (38), one has

(47) $$\begin{array}{cc} dz_{i,n}= & \left({\hat{u}}_{i}-\beta_{i,n}-\Gamma_{i,n}-\Phi_{i,n}\right)dt+{℧}_{i,n}d\omega , \end{array}$$

where $\beta_{i,n}=\sum_{m=1}^{n-1}\frac{\partial {\hat{\alpha }}_{i,n-1}}{\partial {\hat{Y}}_{i,am}}{\dot{\hat{Y}}}_{i,am}+\sum_{m=1}^{n-1}\frac{\partial {\hat{\alpha }}_{i,n-1}}{\partial {\hat{\Theta }}_{i,m}}{\dot{\hat{\Theta }}}_{i,m}+\sum_{m=1}^{n-1}\frac{\partial {\hat{\alpha }}_{i,n-1}}{\partial {\hat{\Theta }}_{si,m}}{\dot{\hat{\Theta }}}_{si,m}+\sum_{m=1}^{n-1}\frac{\partial {\hat{\alpha }}_{i,n-1}}{\partial {\hat{\Theta }}_{pi,m}}{\dot{\hat{\Theta }}}_{pi,m}-\frac{\partial {\hat{\alpha }}_{i,n-1}}{\partial z_{i,1}}\varphi_{i}\beta_{i,1},\Gamma_{i,n}=\sum_{m=1}^{n-1}\frac{\partial {\hat{\alpha }}_{i,n-1}}{\partial x_{i,m}}x_{i,m+1}-f_{i,n}+\;\frac{1}{2}(\frac{\partial^{2}{\hat{\alpha }}_{i,n-1}}{\partial z_{i,1}^{2}}\varphi_{i}^{2}b_{i}^{2}\varrho_{i}^{2}g_{i,1}^{T}g_{i,1}+\sum_{p,q=1}^{n-1}\frac{\partial^{2}{\hat{\alpha }}_{i,n-1}}{\partial x_{i,p}\partial x_{i,q}}g_{i,p}^{T}g_{i,q}$ $+2\sum_{m=1}^{n-1}\frac{\partial^{2}{\hat{\alpha }}_{i,n-1}}{\partial z_{i,1}\partial x_{i,m}}\varphi_{i}b_{i}\varrho_{i}g_{i,m}^{T}g_{i,1})+\frac{\partial {\hat{\alpha }}_{i,n-1}}{\partial z_{i,1}}\varphi_{i}\Gamma_{i,1}+\frac{\partial {\hat{\alpha }}_{i,n-1}}{\partial z_{i,1}}\varphi_{i}F_{si}+\frac{\partial {\hat{\alpha }}_{i,n-1}}{\partial z_{i,1}}\varphi_{i}F_{pi}+\sum_{m=1}^{n-1}\frac{\partial {\hat{\alpha }}_{i,n-1}}{\partial x_{i,m}}f_{i,m}$, $\Phi_{i,n}=\frac{\partial {\hat{\alpha }}_{i,n-1}}{\partial z_{i,1}}\varphi_{i}\left(d_{i}+b_{i}\right)x_{i,n}$ and ${℧}_{i,n}=g_{i,n}-\sum_{m=1}^{n-1}\frac{\partial {\hat{\alpha }}_{i,n-1}}{\partial x_{i,m}}g_{i,m}-\frac{\partial {\hat{\alpha }}_{i,n-1}}{\partial z_{i,1}}{℧}_{i,1}$.

Similar to (16) and (17), define an optimal value function as follows:

(48) $$\begin{array}{cc} J_{i,n}^{*}\left(z_{i,n}\right) & =\lim_{t_{f}\to \infty }\frac{1}{t_{f}}\int_{t}^{t_{f}}W_{i,n}^{*}d\tau , \end{array}$$

where $W_{i,n}^{*}=z_{i,n}^{4}+u_{i}^{*2}$.

Similar to (18)–(24), one has

(49) $$\begin{array}{cc} \frac{\partial V_{i,n}^{*}\left(z_{i,n}\right)}{\partial z_{i,n}}= & 2\left(\eta_{i,n}+\frac{1}{2}{\left(\frac{\partial {\hat{\alpha }}_{i,n-1}}{\partial z_{i,1}}\right)}^{2}\varphi_{i}^{2}\right)z_{i,n}^{3}+2\left({\bar{\eta }}_{i,n}+\delta^{2}+\frac{3}{4}{\bar{\varepsilon }}_{i,n}^{\frac{4}{3}}{\bar{\Phi }}_{i,n}\right)z_{i,n}\\ & +\frac{\phi_{fi,n}^{T}\phi_{fi,n}}{\pi^{2}}z_{i,n}^{3}\Theta_{i,n}^{*}-2\beta_{i,n}+V_{i,n}^{c}, \end{array}$$

(50) $$\begin{array}{cc} u_{i}^{*}= & -\{\left(\eta_{i,n}+\frac{1}{2}{\left(\frac{\partial {\hat{\alpha }}_{i,n-1}}{\partial z_{i,1}}\right)}^{2}\varphi_{i}^{2}\right)z_{i,n}^{3}+({\bar{\eta }}_{i,n}+\frac{3}{4}{\bar{\varepsilon }}_{i,n}^{\frac{4}{3}}{\bar{\Phi }}_{i,n}\\ & +\delta^{2})z_{i,n}+\frac{1}{2}V_{i,n}^{c}+\frac{\phi_{fi,n}^{T}\phi_{fi,n}}{2\pi^{2}}z_{i,n}^{3}\Theta_{i,n}^{*}-\beta_{i,n}\}, \end{array}$$

where $\eta_{i,n}>0,{\bar{\eta }}_{i,n}>0$, $\Theta_{i,n}^{*}$ is the ideal weight, which will be defined later. $V_{i,n}^{c}\left(z_{i,n}\right)=\frac{\partial V_{i,n}^{*}\left(z_{i,n}\right)}{\partial z_{i,n}}-2\left(\eta_{i,n}+\frac{1}{2}{\left(\frac{\partial {\hat{\alpha }}_{i,n-1}}{\partial z_{i,1}}\right)}^{2}\varphi_{i}^{2}\right)z_{i,n}^{3}-2\left({\bar{\eta }}_{i,n}+\delta^{2}+\frac{3}{4}{\bar{\varepsilon }}_{i,n}^{\frac{4}{3}}{\bar{\Phi }}_{i,n}\right)z_{i,n}-\frac{\phi_{fi,n}^{T}\phi_{fi,n}}{\pi^{2}}z_{i,n}^{3}\Theta_{i,n}^{*}+2\beta_{i,n}$.

Similar to (26)–(28), one has

(51) $$\begin{array}{cc} \frac{\partial {\hat{V}}_{i,n}\left(z_{i,n}\right)}{\partial z_{i,n}}= & 2\left(\eta_{i,n}+\frac{1}{2}{\left(\frac{\partial {\hat{\alpha }}_{i,n-1}}{\partial z_{i,1}}\right)}^{2}\varphi_{i}^{2}\right)z_{i,n}^{3}-2\beta_{i,n}+{\hat{Y}}_{i,cn}^{T}\phi_{i,n}\left(z_{i,n}\right)\\ & +\frac{\phi_{fi,n}^{T}\phi_{fi,n}}{\pi^{2}}z_{i,n}^{3}{\hat{\Theta }}_{i,n}+2\left({\bar{\eta }}_{i,n}+\delta^{2}+\frac{3}{4}{\bar{\varepsilon }}_{i,n}^{\frac{4}{3}}{\bar{\Phi }}_{i,n}\right)z_{i,n}, \end{array}$$

(52) $$\begin{array}{cc} {\hat{u}}_{i}= & -\{\left(\eta_{i,n}+\frac{1}{2}{\left(\frac{\partial {\hat{\alpha }}_{i,n-1}}{\partial z_{i,1}}\right)}^{2}\varphi_{i}^{2}\right)z_{i,n}^{3}+({\bar{\eta }}_{i,n}+\frac{3}{4}{\bar{\varepsilon }}_{i,n}^{\frac{4}{3}}{\bar{\Phi }}_{i,n}\\ & +\delta^{2})z_{i,n}+\frac{1}{2}{\hat{Y}}_{i,an}^{T}\phi_{i,n}\left(z_{i,n}\right)+\frac{\phi_{fi,n}^{T}\phi_{fi,n}}{2\pi^{2}}z_{i,n}^{3}{\hat{\Theta }}_{i,n}-\beta_{i,n}\}. \end{array}$$

Similar to (29)–(37), design the adaptive laws ${\dot{\hat{\Theta }}}_{i,n}$, ${\dot{\hat{Y}}}_{i,an}$ and ${\dot{\hat{Y}}}_{i,cn}$ as

(53) $$\begin{array}{c} {\dot{\hat{\Theta }}}_{i,n}=z_{i,n}^{6}\frac{\gamma_{i,n}}{2\pi^{2}}\phi_{fi,n}^{T}\phi_{fi,n}-\sigma_{i,n}{\hat{\Theta }}_{i,n}, \end{array}$$

(54) $$\begin{array}{c} {\dot{\hat{Y}}}_{i,an}=-\gamma_{i,an}\varpi_{i,n}\left({\hat{Y}}_{i,an}-{\hat{Y}}_{i,cn}\right), \end{array}$$

(55) $$\begin{array}{cc} {\dot{\hat{Y}}}_{i,cn}= & -\gamma_{i,cn}\varpi_{i,n}{\hat{Y}}_{i,cn}-\frac{1}{2}\phi_{i,n}\left(z_{i,n}\right)z_{i,n}^{3}, \end{array}$$

where $\varpi_{i,n}=\phi_{i,n}\left(z_{i,n}\right)\phi_{i,n}^{T}\left(z_{i,n}\right)+j_{i,n}I$, $j_{i,n}$, $\gamma_{i,n}$ and $\sigma_{i,n}$ are the design positive constants, $\gamma_{i,an}>0$ and $\gamma_{i,cn}>0$ are the learning rates, and $\gamma_{i,an}>\gamma_{i,cn}$.

**Figure 1:**
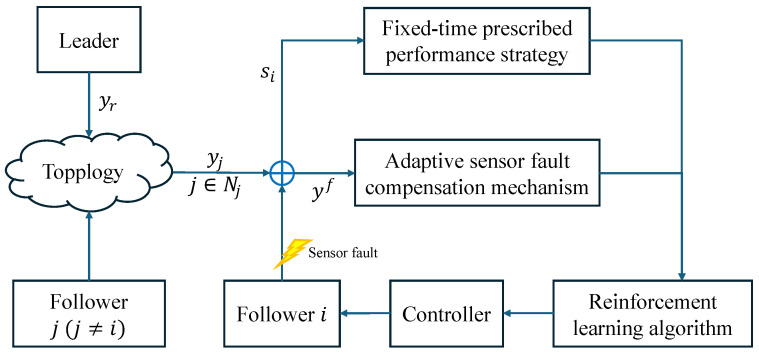
Block diagram of the overall control system.

To clearly demonstrate our ideas and process, a block diagram is provided using Figure 1 and a pseudocode as Algorithm 1.

 **Algorithm 1:** The Fixed-time prescribed performance optimization consensus control algorithm 
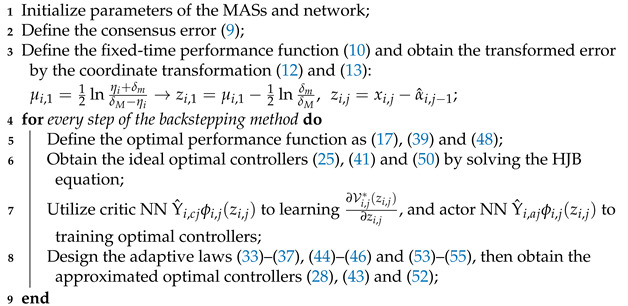


### 3.2. Stability Analysis

**Theorem** **1.** *Consider the SNMASs (3), which satisfies Assumption A1, by designing the local ideal control laws (52), local ideal virtual control law (28) and (43), adaptive laws (33)–(37), (44)–(46), and (53)–(55), the consensus error satisfies the FTPPC requirement, while other signals remain probabilistically bounded.*□

**Proof.**  Define the Lyapunov function for SNMASs (3) as

(56) $$\begin{array}{cc} V= & \sum_{i=1}^{N}\sum_{m=1}^{n}V_{i,m}, \end{array}$$

where $V_{i,1}=\frac{1}{4}z_{i,1}^{4}+\frac{1}{2\gamma_{i,1}}{\tilde{\Theta }}_{i,1}^{2}+\frac{1}{2{\bar{\gamma }}_{i,1}}{\tilde{\Theta }}_{si,1}^{2}+\frac{1}{2{\underline{\gamma }}_{i,1}}{\tilde{\Theta }}_{pi,1}^{2}+\frac{1}{2}{\tilde{Y}}_{i,a1}^{T}{\tilde{Y}}_{i,a1}+\frac{1}{2}{\tilde{Y}}_{i,c1}^{T}{\tilde{Y}}_{i,c1}$, $V_{i,j}=\frac{1}{4}z_{i,j}^{4}+\frac{1}{2\gamma_{i,j}}{\tilde{\Theta }}_{i,j}^{2}+\frac{1}{2}{\tilde{Y}}_{i,aj}^{T}{\tilde{Y}}_{i,aj}+\frac{1}{2}{\tilde{Y}}_{i,cj}^{T}{\tilde{Y}}_{i,cj},j=2,\ldots ,n$, ${\tilde{Y}}_{i,am}={\hat{Y}}_{i,am}-Y_{i,m}^{*}$ and ${\tilde{Y}}_{i,cm}={\hat{Y}}_{i,cm}-Y_{i,m}^{*}$. ${\tilde{\Theta }}_{i,m}={\hat{\Theta }}_{i,m}-\Theta_{i,m}^{*}$ with $\Theta_{i,m}^{*}={∥Y_{i,m}^{*}∥}^{2}$, m=1,…,n, ${\tilde{\Theta }}_{si,1}={\hat{\Theta }}_{si,1}-\Theta_{si,1}^{*}$ with $\Theta_{si,1}^{*}={∥Y_{si,1}^{*}∥}^{2}$, and ${\tilde{\Theta }}_{pi,1}={\hat{\Theta }}_{pi,1}-\Theta_{pi,1}^{*}$ with $\Theta_{pi,1}^{*}={∥Y_{pi}^{*}∥}^{2}$.

From (5), (13), (33) and (34), one has $LV_{i,1}$ is

(57) $$\begin{array}{cc} LV_{i,1}= & z_{i,1}^{3}\varphi_{i}\left[\left(d_{i}+b_{i}\right)\left(z_{i,2}+{\hat{\alpha }}_{i,1}\right)+F_{si}+F_{pi}-\beta_{i,1}+\Gamma_{i,1}\right]-\frac{\gamma_{i,a1}}{{\left(d_{i}+b_{i}\right)}^{2}}{\tilde{Y}}_{i,a1}^{T}\varpi_{i,1}({\hat{Y}}_{i,a1}\\ & -{\hat{Y}}_{i,c1})+\frac{3z_{i,1}^{2}}{2}{∥{℧}_{i,1}∥}^{2}+\frac{{\tilde{\Theta }}_{i,1}}{\gamma_{i,1}}{\dot{\hat{\Theta }}}_{i,1}+\frac{{\tilde{\Theta }}_{si,1}}{\gamma_{si,1}}{\dot{\hat{\Theta }}}_{si,1}+\frac{{\tilde{\Theta }}_{pi,1}}{\gamma_{pi,1}}{\dot{\hat{\Theta }}}_{pi,1}\\ & -\frac{\varphi_{i}}{2}{\tilde{Y}}_{i,c1}^{T}\phi_{i,1}\left(z_{i,1}\right)z_{i,1}^{3}-\frac{\gamma_{i,c1}}{{\left(d_{i}+b_{i}\right)}^{2}}{\tilde{Y}}_{i,c1}^{T}\varpi_{i,1}{\hat{Y}}_{i,c1}. \end{array}$$

By applying (7), one has

(58) $$\begin{array}{c} \frac{3z_{i,1}^{2}\varphi_{i}^{2}}{2}∥{℧}_{i,1}{∥}^{2}\leq \frac{9}{\varsigma^{3}}+\frac{\varsigma^{3/2}z_{i,1}^{3}\varphi_{i}^{3}}{3\sqrt{2}}{∥b_{i}\varrho_{i}g_{i,1}∥}^{3}, \end{array}$$

where ς>0 is a constant.

Define $F_{i,1}\left(Z_{i,1}\right)=\frac{\varsigma^{3/2}\varphi_{i}^{2}}{3\sqrt{2}}{∥b_{i}\varrho_{i}g_{i,1}∥}^{3}+\Gamma_{i,1}$ with $Z_{i,1}={\left[z_{i,1},x_{i,1},{\bar{x}}_{k,2}\right]}^{T}$, similar to (26), we can express $F_{i,1}\left(Z_{i,1}\right)$ as follows

(59) $$\begin{array}{c} F_{i,1}\left(Z_{i,1}\right)=Y_{fi,1}^{*T}\phi_{fi,1}\left(Z_{i,1}\right)+\varepsilon_{fi,1}\left(Z_{i,1}\right), \end{array}$$

where $∥\varepsilon_{i,1}\left(Z_{i,1}\right)∥\leq \varepsilon_{i,1}^{*}$ with constant $\varepsilon_{i,1}^{*}>0$.

By applying (7), one has

(60) $$\begin{array}{cc} z_{i,1}^{3}\varphi_{i}Y_{m}^{*T}\phi_{m} & \leq z_{i,1}^{6}\varphi_{i}^{2}\frac{\Theta_{m,1}^{*}}{2\pi^{2}}\phi_{m}^{T}\phi_{m}+\frac{\pi^{2}}{2},\;m=si,pi,\left(fi,1\right), \end{array}$$

(61) $$\begin{array}{c} z_{i,1}^{3}\varphi_{i}\varepsilon_{m}\leq \frac{1}{4}z_{i,1}^{6}\varphi_{i}^{2}+\varepsilon_{m}^{*2},\;m=si,pi,\left(fi,1\right), \end{array}$$

(62) $$\begin{array}{c} z_{i,1}^{3}\varphi_{i}\left(d_{i}+b_{i}\right)z_{i,2}\leq \frac{1}{4}z_{i,1}^{6}\varphi_{i}^{2}+{\left(d_{i}+b_{i}\right)}^{2}z_{i,2}^{2}, \end{array}$$

(63) $$\begin{array}{c} {\left(d_{i}+b_{i}\right)}^{2}z_{i,2}^{2}\leq \frac{1}{4\delta_{i,1}^{2}}{\left(d_{i}+b_{i}\right)}^{4}+\delta_{i,1}^{2}z_{i,2}^{4}, \end{array}$$

(64) $$\begin{array}{c} {\tilde{Y}}_{i,a1}^{T}\varpi_{i,1}{\tilde{Y}}_{i,c1}\leq \frac{1}{2}{\tilde{Y}}_{i,a1}^{T}\varpi_{i,1}{\tilde{Y}}_{i,a1}+\frac{1}{2}{\tilde{Y}}_{i,c1}^{T}\varpi_{i,1}{\tilde{Y}}_{i,c1}, \end{array}$$

(65) $$\begin{array}{c} -\frac{1}{2}Y^{T}\phi_{i,1}\varphi_{i}z_{i,1}^{3}\leq \frac{1}{4}Y_{i,a1}^{T}\varpi_{i,1}Y+\frac{1}{4}\varphi_{i}^{2}z_{i,1}^{6},\;Y={\tilde{Y}}_{i,a1},{\hat{Y}}_{i,c1}, \end{array}$$

(66) $$\begin{array}{cc} -\frac{\sigma_{m,1}}{\gamma_{m,1}}{\tilde{\Theta }}_{m,1}{\hat{\Theta }}_{m,1}\leq & \frac{\sigma_{m,1}}{2\gamma_{m,1}}\Theta_{m,1}^{*2}-\frac{\sigma_{m,1}}{2\gamma_{m,1}}{\tilde{\Theta }}_{m,1}^{2},\;m=i,si,pi. \end{array}$$

Then, the following equations hold:

(67) $$\begin{array}{c} {\hat{Y}}_{i,a1}-{\hat{Y}}_{i,c1}={\tilde{Y}}_{i,a1}-{\tilde{Y}}_{i,c1}, \end{array}$$

(68) $$\begin{array}{cc} & -\frac{\varphi_{i}}{2}z_{i,1}^{3}{\hat{Y}}_{i,a1}^{T}\phi_{i,1}-\frac{\varphi_{i}}{2}z_{i,1}^{3}{\tilde{Y}}_{i,c1}^{T}\phi_{i,1}=-\frac{\varphi_{i}}{2}z_{i,1}^{3}{\tilde{Y}}_{i,a1}^{T}\phi_{i,1}-\frac{\varphi_{i}}{2}z_{i,1}^{3}{\hat{Y}}_{i,c1}^{T}\phi_{i,1}, \end{array}$$

(69) $$\begin{array}{cc} {\tilde{Y}}_{i,c1}^{T}\varpi_{i,1}{\hat{Y}}_{i,c1}= & \frac{1}{2}{\tilde{Y}}_{i,c1}^{T}\varpi_{i,1}{\tilde{Y}}_{i,c1}+\frac{1}{2}{\hat{Y}}_{i,c1}^{T}\varpi_{i,1}{\hat{Y}}_{i,c1}-\frac{1}{2}Y_{fi,1}^{*T}\varpi_{i,1}Y_{fi,1}^{*}. \end{array}$$

By invoking (35)–(37) and (57)–(69) yields

(70) $$\begin{array}{cc} LV_{i,1}\leq & -\left(\eta_{i,1}-\frac{3}{2}\right)\varphi_{i}^{2}z_{i,1}^{6}-\frac{\gamma_{i,c1}-\gamma_{i,a1}}{2{\left(d_{i}+b_{i}\right)}^{2}}{\tilde{Y}}_{i,c1}^{T}\varpi_{i,1}{\tilde{Y}}_{i,c1}-{\bar{\eta }}_{i,1}z_{i,1}^{4}-\frac{\sigma_{i,1}}{2\gamma_{i,1}}{\tilde{\Theta }}_{i,1}^{2}\\ & -\frac{\sigma_{si,1}}{2\gamma_{si,1}}{\tilde{\Theta }}_{si,1}^{2}-\frac{\sigma_{pi,1}}{2\gamma_{pi,1}}{\tilde{\Theta }}_{pi,1}^{2}-\left(\frac{\gamma_{i,c1}}{2{\left(d_{i}+b_{i}\right)}^{2}}-\frac{1}{4}\right){\hat{Y}}_{i,c1}^{T}\varpi_{i,1}{\hat{Y}}_{i,c1}+\Delta_{i,1}\\ & +\delta_{i,1}^{2}z_{i,2}^{4}-\left(\frac{\gamma_{i,a1}}{2{\left(d_{i}+b_{i}\right)}^{2}}-\frac{1}{4}\right){\tilde{Y}}_{i,a1}^{T}\varpi_{i,1}{\tilde{Y}}_{i,a1}, \end{array}$$

where $\Delta_{i,1}=\varepsilon_{fi,1}^{*2}+\varepsilon_{si}^{*2}+\varepsilon_{pi}^{*2}+\frac{\pi^{2}}{2}+\frac{\pi^{2}}{2}+\frac{\pi^{2}}{2}+\frac{\sigma_{i,1}}{2\gamma_{i,1}}\Theta_{i,1}^{*2}+\frac{\sigma_{si,1}}{2\gamma_{si,1}}\Theta_{si,1}^{*2}+\frac{\sigma_{pi,1}}{2\gamma_{pi,1}}\Theta_{pi,1}^{*2}+\frac{9}{\varsigma^{3}}+\frac{1}{4\delta^{2}}{\left(d_{i}+b_{i}\right)}^{4}+\frac{\gamma_{i,c1}}{2{\left(d_{i}+b_{i}\right)}^{2}}Y_{fi,1}^{*T}\varpi_{i,1}Y_{fi,1}^{*}$.

Let $\eta_{i,1}>\frac{3}{2}$, $\gamma_{i,a1}>\frac{{\left(d_{i}+b_{i}\right)}^{2}}{2}$, $\gamma_{i,c1}>\gamma_{i,a1}$, $\gamma_{i,1}^{*}=\min\left\{\frac{\gamma_{i,c1}-\gamma_{i,a1}}{{\left(d_{i}+b_{i}\right)}^{2}},\frac{\gamma_{i,a1}}{{\left(d_{i}+b_{i}\right)}^{2}}-\frac{1}{2}\right\}$, $c_{i,1}=\min\left\{4{\bar{\eta }}_{i,1},\sigma_{i,1},\sigma_{si},\sigma_{pi},\frac{\gamma_{i,1}^{*}\lambda_{\min(i,1)}}{{\left(d_{i}+b_{i}\right)}^{2}}\right\}$, $\lambda_{\min(i,1)}$ is the minimal characteristic value of $\varpi_{i,1}$, rewrite (70) as

(71) $$\begin{array}{c} LV_{i,1}\leq -c_{i,1}V_{i,1}+\Delta_{i,1}+\delta_{i,1}^{2}z_{i,2}^{4}. \end{array}$$

By applying (7), one has

(72) $$\begin{array}{c} z_{i,j}^{3}\varepsilon_{i,j}\leq \frac{1}{2}z_{i,j}^{6}+\frac{1}{2}\varepsilon_{fi,j}^{*2}, \end{array}$$

(73) $$\begin{array}{c} z_{i,j}^{3}z_{i,j+1}\leq \frac{1}{2}z_{i,j}^{6}+\frac{1}{2}z_{i,j+1}^{2}, \end{array}$$

(74) $$\begin{array}{c} \frac{1}{2}z_{i,j+1}^{2}\leq \delta^{2}z_{i,j+1}^{4}+\frac{1}{16\delta^{2}}, \end{array}$$

(75) $$\begin{array}{c} -z_{i,j}^{3}\Phi_{i,j}\leq \frac{3}{4}{\bar{\varepsilon }}_{i,j}^{\frac{4}{3}}z_{i,j}^{4}\Phi_{i,j}^{\frac{4}{3}}+\frac{1}{4{\bar{\varepsilon }}_{i,j}^{4}}. \end{array}$$

Similar to $LV_{i,1}$, one has

(76) $$\begin{array}{cc} LV_{i,j}\leq & -\left(\eta_{i,j}-\frac{3}{2}\right)z_{i,j}^{6}+\delta^{2}\left(z_{i,j+1}^{4}-z_{i,j}^{4}\right)-\frac{\sigma_{i,j}}{2\gamma_{i,j}}{\tilde{\Theta }}_{i,j}^{2}-\frac{\gamma_{i,cj}-\gamma_{i,aj}}{2}{\tilde{Y}}_{i,cj}^{T}\varpi_{i,j}{\tilde{Y}}_{i,cj}\\ & -{\bar{\eta }}_{i,j}z_{i,j}^{4}-\left(\frac{\gamma_{i,cj}}{2}-\frac{1}{4}\right){\hat{Y}}_{i,cj}^{T}\varpi_{i,j}{\hat{Y}}_{i,cj}-\left(\frac{\gamma_{i,aj}}{2}-\frac{1}{4}\right){\tilde{Y}}_{i,aj}^{T}\varpi_{i,j}{\tilde{Y}}_{i,aj}+\Delta_{i,j}, \end{array}$$

where $\Delta_{i,j}=\frac{\sigma_{i,j}}{2\gamma_{i,j}}\Theta_{i,j}^{*2}+\frac{9}{\varsigma^{3}}+\frac{\pi^{2}}{2}+\frac{1}{2}\varepsilon_{fi,j}^{*2}+\frac{1}{4{\bar{\varepsilon }}_{i,j}^{4}}+\frac{1}{16\delta^{2}}+\frac{\gamma_{i,cj}}{2}Y_{fi,j}^{*T}\varpi_{i,j}Y_{fi,j}^{*}$.

Let $\eta_{i,j}>\frac{3}{2},\gamma_{i,aj}>\frac{1}{2}$, $\gamma_{i,cj}>\gamma_{i,aj}$, $\gamma_{i,j}^{*}=\min\left\{\gamma_{i,cj}-\gamma_{i,aj},\gamma_{i,aj}-\frac{1}{2}\right\}$, $c_{i,j}=\min\{4{\bar{\eta }}_{i,j},$ $\sigma_{i,j},\sigma_{si},\sigma_{pi},\gamma_{i,j}^{*}\lambda_{\min(i,j)}\}$, $\lambda_{\min(i,j)}$ is the minimal characteristic value of $\varpi_{i,j}$, rewrite (76) as

(77) $$\begin{array}{cc} LV_{i,j}\leq & -c_{i,j}V_{i,j}+\Delta_{i,j}+\delta^{2}\left(z_{i,j+1}^{4}-z_{i,j}^{4}\right). \end{array}$$

Similar to $LV_{i,j}$, one has

(78) $$\begin{array}{cc} LV_{i,n}\leq & -\left(\eta_{i,n}-\frac{3}{4}\right)z_{i,n}^{6}-{\bar{\eta }}_{i,n}z_{i,n}^{4}-\delta^{2}z_{i,n}^{4}-\frac{\sigma_{i,n}}{2\gamma_{i,n}}{\tilde{\Theta }}_{i,n}^{2}-\frac{\gamma_{i,cn}-\gamma_{i,an}}{2}{\tilde{Y}}_{i,cn}^{T}\varpi_{i,n}{\tilde{Y}}_{i,cn}\\ & +\Delta_{i,n}-\left(\frac{\gamma_{i,cn}}{2}-\frac{1}{4}\right){\hat{Y}}_{i,cn}^{T}\varpi_{i,n}{\hat{Y}}_{i,cn}-\left(\frac{\gamma_{i,an}}{2}-\frac{1}{4}\right){\tilde{Y}}_{i,an}^{T}\varpi_{i,n}{\tilde{Y}}_{i,an}, \end{array}$$

where $\Delta_{i,n}=\frac{\sigma_{i,n}}{2\gamma_{i,n}}\Theta_{i,n}^{*2}+\frac{9}{\varsigma^{3}}+\frac{\pi^{2}}{2}+\frac{\gamma_{i,cj}}{2}Y_{fi,n}^{*T}\varpi_{fi,n}Y_{i,n}^{*}+\frac{1}{2}\varepsilon_{fi,n}^{*2}+\frac{1}{4{\bar{\varepsilon }}_{i,n}^{4}}$.

Let $\eta_{i,n}>\frac{3}{4},\gamma_{i,an}>\frac{1}{2}$ and $\gamma_{i,cn}>\gamma_{i,an}$, $\gamma_{i,n}^{*}=\min\left\{\gamma_{i,cn}-\gamma_{i,an},\gamma_{i,an}-\frac{1}{2}\right\}$, $\lambda_{\min(i,n)}$ is the minimal characteristic value of $\varpi_{i,n}$, $c_{i,n}=\min\left\{4{\bar{\eta }}_{i,n},\sigma_{i,n},\sigma_{si},\sigma_{pi},\gamma_{i,n}^{*}\lambda_{\min(i,n)}\right\}$, rewrite (78) as

(79) $$\begin{array}{cc} LV_{i,n}\leq & -c_{i,n}V_{i,n}+\Delta_{i,n}-\delta^{2}z_{i,n}^{4}. \end{array}$$

From (56), (71), (77), and (79), one has

(80) LV≤−CV+Δ,

where $C=\min_{1\leq i\leq N,1\leq m\leq n,2\leq j\leq n}\{4{\bar{\eta }}_{i,m},\sigma_{i,m},\sigma_{si,m},\sigma_{pi,m},$$\frac{\gamma_{i,1}^{*}\lambda_{\min(i,1)}}{{\left(d_{i}+b_{i}\right)}^{2}},\gamma_{i,j}^{*}\lambda_{\min(i,j)}\}$, $\Delta =\sum_{i=1}^{N}\sum_{m=1}^{n}\Delta_{i,m}$.

Based on $It\hat{o}$ Lemma and (80), one has

(81) $$E\left[V\left(t\right)\right]\leq V\left(t_{0}\right)e^{-C(t-t_{0})}+\frac{\Delta }{C}.$$

Therefore, it can be concluded that Theorem 1 holds true based on the above analysis.

## 4. Simulation Example

**Figure 2:**
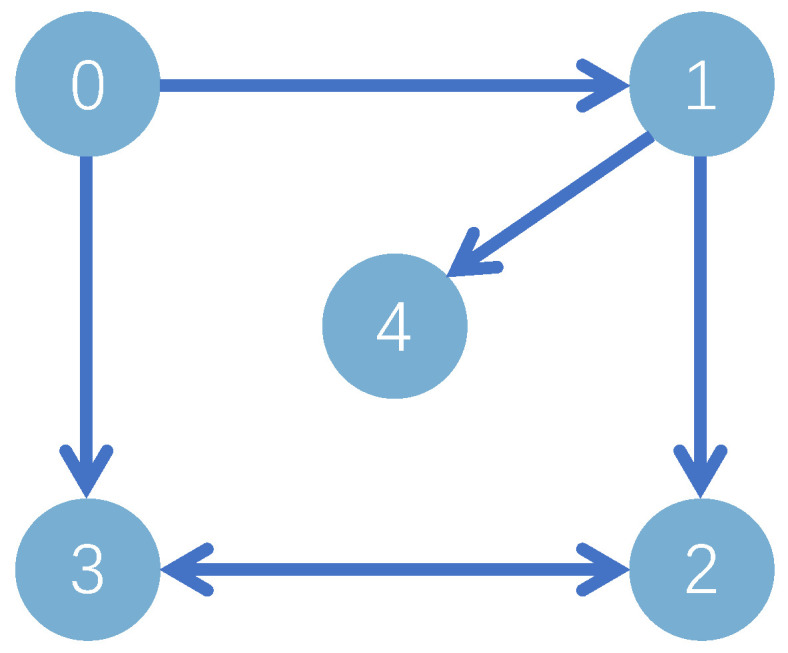
Directed communication topology graph.

Consider the following SNMASs, with the directed communication topology graph Figure 2.

(82) $$\left\{\begin{array}{cc} dx_{i,1} & =\left(x_{i,2}+\cos\left(x_{i,1}\right)\right)dt+\sin\left(6x_{i,1}\right)d\omega \\ dx_{i,2} & =\left(u_{i}+\cos\left(x_{i,1}\right)\sin\left(5x_{i,2}\right)\right)dt+\sin\left(6x_{i,1}x_{i,2}\right)d\omega \\ y_{i}^{f} & =\varrho_{i}x_{i,1}+\rho_{i},1\leq i\leq 4, \end{array}\right.$$

The desired trajectory is $y_{r}=\frac{1}{2}\left(\sin\left(5t\right)+1\right)$. From Figure 2, we have

$$A=\left[\begin{array}{ccccccc} 0 & & 0 & & 0 & & 0\\ 1 & & 0 & & 1 & & 0\\ 0 & & 1 & & 0 & & 0\\ 1 & & 0 & & 0 & & 0 \end{array}\right],L=\left[\begin{array}{ccccccc} 0 & & 0 & & 0 & & 0\\ -1 & & 2 & & -1 & & 0\\ 0 & & -1 & & 1 & & 0\\ -1 & & 0 & & 0 & & 1 \end{array}\right].$$

Then, the FTPF is chosen as

$$\begin{array}{c} h\left(t\right)=\left\{\begin{array}{cc} 4.9{\left(1-4t\right)}^{2}+0.1, & 0\leq t<0.25\\ 0.1, & t\geq 0.25, \end{array}\right. \end{array}$$

from which we can obtain $h_{0}=5,h_{\tilde{T}}=0.1,\iota =2,\lambda =8$, and $\tilde{T}=0.25$.

**Figure 3:**
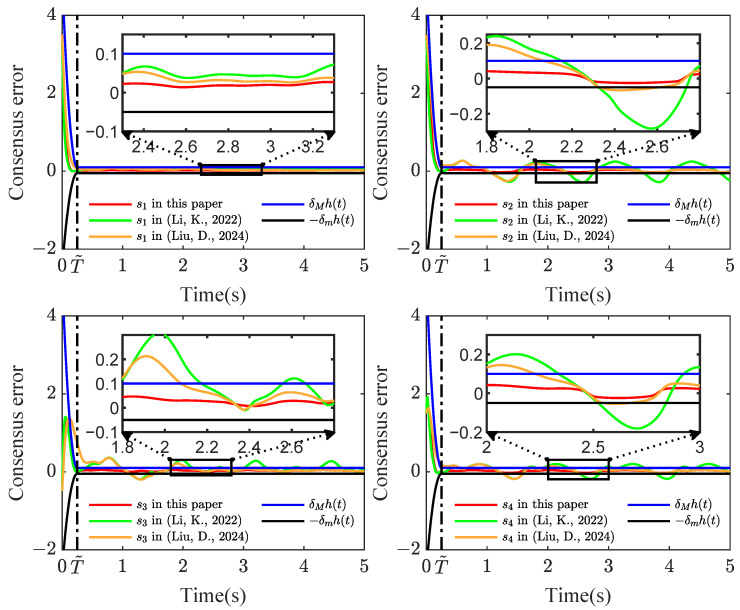
Schematic diagram of $s_{i}\left(t\right)$ curves [44,46].

**Figure 4:**
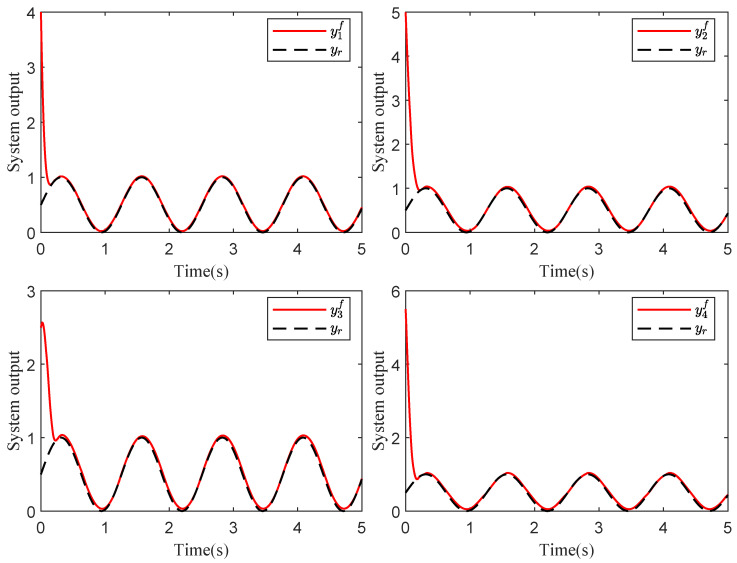
Schematic diagram of $y_{i}^{f}$ and $y_{r}$ curves.

**Figure 5:**
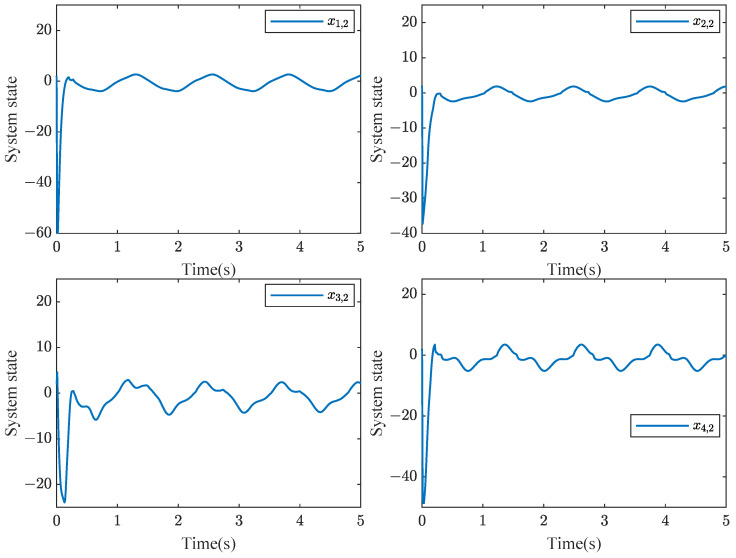
Schematic diagram of $x_{i,2}$ curvies.

**Figure 6:**
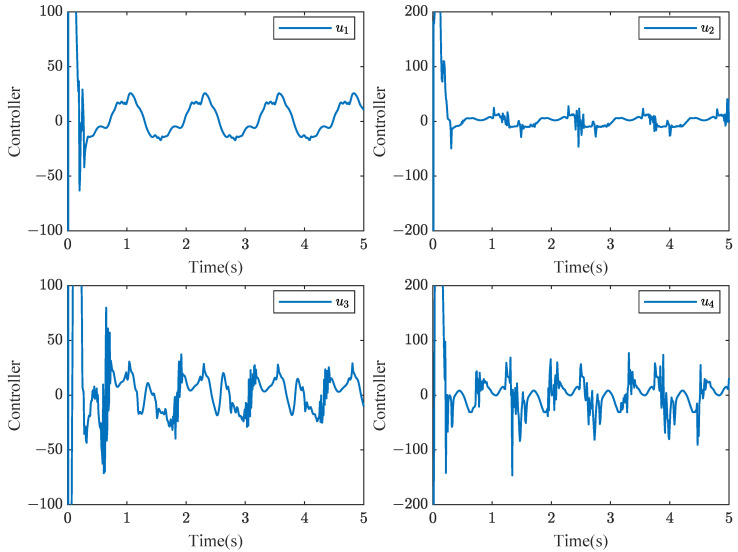
Schematic diagram of $u_{i}\left(t\right)$ curvies.

Based on stability analysis, we take the design parameters as $\eta_{i,1}=\eta_{i,2}=1.6$,${\bar{\eta }}_{i,1}={\bar{\eta }}_{i,2}=20$, $\mu_{i,1}={\left[-2,-1,0,1,2\right]}^{T}$, $\mu_{i,2}={\left[-3,-2,-1,0,1,2,3\right]}^{T}$, $\sigma_{i}=1$, $\sigma_{i,1}=\sigma_{si,1}=\sigma_{pi,1}=\gamma_{i,c1}=\gamma_{i,c2}=\gamma_{i,1}=\gamma_{si,1}=\gamma_{pi,1}=15$, $\sigma_{i,2}=\sigma_{si,2}=\sigma_{pi,2}=\gamma_{i,2}=\gamma_{si,2}=\gamma_{pi,2}=10$, $\gamma_{i,a1}=\gamma_{i,a2}=5$, $\delta_{m}=0.5$, $\delta_{M}=1$. The initial values are given by $x_{i,1}\left(0\right)=5$, $x_{i,2}\left(0\right)=2$, $Y_{i,c1}\left(0\right)=0.5$, $Y_{i,a1}\left(0\right)=0.55$, $Y_{i,c2}\left(0\right)=0.8$, $Y_{i,a2}\left(0\right)=0.85$, $\Theta_{i,1}\left(0\right)=\Theta_{i,2}\left(0\right)=\Theta_{si,1}\left(0\right)=\Theta_{si,2}\left(0\right)=\Theta_{pi,1}\left(0\right)=\Theta_{pi,2}\left(0\right)=1$. Consider the sensor faults: drift fault ($\varrho_{1}=0.8,\rho_{1}=0$), time-varying bias fault ($\varrho_{2}=1,\rho_{2}=-\exp\left(-x_{2,1}\right)$), time-varying drift fault ($\varrho_{3}=1/\left(1+\exp\left(-t\right)\right),\rho_{3}=0$), and bias fault ($\varrho_{4}=1,\rho_{4}=0.5$). Simulation results are depicted in Figure 3, Figure 4, Figure 5 and Figure 6.

Figure 3 shows that the consensus error is driven to a target neighborhood that satisfies the FTPPC requirement. Meanwhile, by comparing different control methods, the effectiveness of the implemented control method is proven. Compared with the FTC in [44], the proposed method ensures that the consensus error converges to a prescribed range. In contrast to the PPC in [46], this method is able to converge to the prescribed range in fixed time, resulting in an average time saving of about 88.1657%. Figure 4 shows that under the controller (52), the followers can effectively follow the leader’s trajectory. The controller curves $u_{i}$ are displayed in Figure 6. Figure 4, Figure 5 and Figure 6 show that $x_{i,2}$, $y_{i}^{f}$ and $u_{i}$ are bounded in probability. Undoubtedly, the designed controller has been validated as effective through simulation results.

## 5. Conclusions

For the SNMASs with sensor faults, the fixed-time prescribed performance optimal consensus control issues have been addressed. A control protocol based on inaccurate information has been proposed to address the issue where the existing feedback control law is not applicable under sensor faults. By utilizing RL and backstepping, we realized the FTPPC for the consensus error. Compared with other consensus control methods, the proposed method is able to converge to a prescribed range, and the convergence time is saved by an average of about 88.1657%. Future work will investigate consensus control for MASs with time delays [47] based on the “Data-Driven ToMFIR” technique [48,49]. 

## Data Availability

Data are contained within the article.

## Appendix A

Tables of key variables and abbreviations are provided to help ensure that readers can clearly understand our paper.

**Table A1:** The key variable of this paper.

Variable	Definition
$x_{i,\cdot }$	System state
$y_{i}^{f}$	System output state with sensor fault
*t*	Time
$s_{i}$	Consensus error
$\alpha_{i},\cdot$	Virtual controller
$u_{i}$	Actual controller
$Y_{i,\cdot }$	Network weight
${\left(\cdot \right)}^{*}$	Optimal parameters
$\hat{\left(\cdot \right)}$	Approximate optimal parameters

**Table A2:** The abbreviation of this paper.

Abbreviation	Full Spelling
MASs	Multi-agent systems
SNMASs	Stochastic Nonlinear MASs
FTC	Fixed-time control
FTPPC	Fixed-time prescribed performance control
PPC	Prescribed performance control
HJB	Hamilton–Jacobi–Bellman
RL	Reinforcement learning
NNs	Neural networks
FTPF	Fixed-time performance function
